# Vaccine related crowdfunding on a ‘Freedom Fundraising’ platform

**DOI:** 10.1371/journal.pone.0288539

**Published:** 2023-07-12

**Authors:** Jeremy Snyder, Marco Zenone

**Affiliations:** 1 Faculty of Health Sciences, Simon Fraser University, Burnaby, British Columbia, Canada; 2 Health Law Institute, Faculty of Law, University of Alberta, Edmonton, Alberta, Canada; Al-Zaytoonah University for Science and Technology, STATE OF PALESTINE

## Abstract

**Introduction:**

Donation-based crowdfunding was heavily used during the COVID-19 pandemic. While most of these campaigns were uncontroversial, others spread misinformation or undermined public health. In response, mainstream crowdfunding platforms like GoFundMe restricted what campaigns they would host. This led some campaigns to shift to lesser-known and less restrictive crowdfunding platforms. While research on health-related misinformation on mainstream crowdfunding platforms is increasing, less is known about crowdfunding on less restrictive platforms like GiveSendGo. The aim of this study is to review vaccine-related crowdfunding campaigns on the GiveSendGo platform to better understand: 1) how vaccines are portrayed on GiveSendGo; and 2) how successful these campaigns have been at attracting financial support.

**Methods:**

We searched the GiveSendGo crowdfunding platform for campaigns including “vaccine” or “vaccination”. This process yielded 907 unique results which were then scraped for their campaign text and fundraising data. The authors reviewed these campaigns for fundraisers whose aims related to vaccines for humans and assigned campaigns as being for 1) Accessing vaccines; 2) creating Spaces for the unvaccinated; 3) helping Unvaccinated Individuals); 4) Advocacy about vaccines; 5) supporting Anti-Mandate actions; and 6) responding to Vaccine Injuries.

**Findings:**

We identified 765 crowdfunding campaigns that raised $6,814,817 and requested $838,578,249. Anti-Mandate campaigns were most common, followed by Unvaccinated Individuals, Vaccine Injuries, Advocacy, Access, and Spaces. Only Access campaigns took a positive or neutral view toward vaccines. Themes of freedom and religion cut across campaign types with campaigns critical of vaccines invoking bodily autonomy and religious freedom as justifying their fundraisers.

**Discussion:**

Very few of these fundraisers met their goals. With the exception of Access campaigns, they frequently contained highly polarizing language advocating against public health mandates, misinformation about vaccine safety, and language from bioethics and reproductive choice advocates. Restrictions on vaccine-related campaigns on the GoFundMe platform likely drove campaign creation on GiveSendGo.

## Introduction

Donation-based crowdfunding is a practice where campaign organizers use online platforms to solicit financial help from friends, family, and the wider public. The most common category of donation-based crowdfunding on many platforms is fundraising for health-related needs. The popularity of crowdfunding tied to medical needs is longstanding and also grew sharply during the COVID-19 pandemic [1]. Medical crowdfunding commonly includes raising money to pay for medical care, but ill health can also contribute to loss of income and other indirect costs that can be addressed through crowdfunding. Moreover, as the COVID-19 pandemic demonstrated, a public health emergency can cause a range of non-medical hardships such as loss of income and access to services due to social distancing measures—all of which are targets for crowdfunding campaigns [2].

GoFundMe, the largest donation-based crowdfunding platform in North America, reported that between March 1 and August 31, 2020 more than $625 million was raised for campaigns responding to the COVID-19 pandemic [3]. These topline numbers show a large impact from donation-based crowdfunding; however, the success of individual campaigns is varied and more limited. A study of 164,311 crowdfunding campaigns on the GoFundMe platform that were created in response to the COVID-19 pandemic from January 1 to July 31, 2020 found that they raised over $380 million but that the median campaign raised only $65 out of a goal of $5,000. 43.2% of these campaigns had no donations at all [4].

While crowdfunding campaigns were able to help some recipients during the COVID-19 pandemic, they also raised challenges for crowdfunding platforms. In particular, these companies faced decisions as to what kinds of pandemic-related campaigns they would be willing to host. This is part of a longstanding gatekeeping function for many crowdfunding and other online platforms who are tasked with moderating the content that they host, especially if it includes misinformation or discriminatory and hateful language. This is not an altruistic activity on the part of these largely private companies as allowing fundraisers for antidemocratic groups and people accused of violence and discrimination can lead to some campaigners choosing to take their business to another platform. This is particularly the case with larger charitable institutions who may not want to be associated with politically charged fundraisers [5].

GoFundMe has received a great deal of attention for these gatekeeping choices. For example, it has policies against fundraisers that encourage hate or fund the legal defense of people accused of violent crimes. These policies have led it to remove a number of prominent campaigns for the legal defense of police officers accused of murder, January 6 insurrectionists, and others [6]. In response to fundraisers that included false information about vaccines, GoFundMe instituted a policy in 2019 that prohibited campaigns that contain vaccine misinformation. This policy resulted in hundreds of campaigns being removed during the COVID-19 pandemic and others having resource labels added to them that directed potential donors to vetted information sources on vaccines from the World Health Organization and US Federal Drug Administration [7].

As the pandemic went on and many public and private entities instituted vaccine mandates for employees, customers, and others, individuals began creating crowdfunding campaigns seeking to overturn these mandates or support those negatively impacted by them. By far the largest of these was a campaign first started on GoFundMe to support an anti-vaccine mandate protest in Canada’s capital city, Ottawa. This protest initially focused on a rule change that would require truck drivers crossing the international border with the US to be vaccinated. Soon after, it expanded into a general protest against vaccine mandates and other public health measures [8]. The Freedom Convoy campaign raised over USD $7 million but was initially frozen by GoFundMe due to a lack of clarity as to how funds would be used. After reports of violence and other unlawful activity by protesters, the campaign was removed entirely from GoFundMe [6, 9].

But this was not the end of the story for this fundraiser or many others that have been restricted or removed by GoFundMe and other crowdfunding platforms. Instead, the organizers of the Freedom Convoy fundraiser moved it to the crowdfunding platform GiveSendGo, which brands itself as a Christian platform that is the “Leader in Freedom Fundraising” [10]. In the case of the Freedom Convoy fundraiser, moving to GiveSendGo did not hurt its success as it went on to raise nearly USD $10 million, more money than was raised (and later refunded) on GoFundMe and enough to become one of the largest donation-based crowdfunding campaigns in Canada [11]. Prior to that, GiveSendGo had been host to a range of campaigns raising concerns about the safety of vaccines as well as serving as a home to far-right causes that had been banned from or faced restrictions on GoFundMe and other more mainstream crowdfunding platforms [12, 13].

Crowdfunding campaigns have been found to be significant sources of medical misinformation [14], provide funding for purveyors of unproven medical interventions [15], and contain highly persuasive but misleading personal testimonials [16]. However, studies of crowdfunding campaigns have almost exclusively focused on the largest and most restrictive platforms like GoFundMe. Thus, the aim of this study is to review vaccine-related crowdfunding campaigns on the GiveSendGo platform to better understand: 1) how vaccines are portrayed on GiveSendGo; and 2) how successful these campaigns have been at attracting financial support. This information is important to understanding the effectiveness of content moderation in the donation-based crowdfunding sector and how vaccines and vaccine-related public health policy are being interpreted by the public, especially during the COVID-19 pandemic.

## Methods

The GiveSendGo website was searched using that website’s internal search engine for campaigns that included the words “vaccine” or “vaccination”. This search was conducted on September 19, 2022 and produced 907 unique results. The URLs from these results were then scraped using an automated web scraping tool on September 20 and October 5, 2022 to retrieve the campaign title, campaign text, update text, funding requested, funding received, the URL of any uploaded images, and the number of donors, shares, and prayers from each campaign page. This process complied with the terms and conditions of the software used for data scraping. Consent was not sought for this study as these campaigns were publicly posted and intended to be viewed by the public and disseminated via social media. As this data is publicly available and posted without an expectation of privacy, research ethics approval for this study was not required.

The URLs of uploaded images, when available, include an upload date that implies a creation date for the campaign. This information was converted by the first author to creation dates for each campaign. When donation and goal totals were not recorded in US dollars, these currencies were converted by the first author to US dollars using the exchange rate on the day the campaign was created. Non-US dollar donation and goal totals were then used to identify the country location of campaigns outside of the US.

The scraped campaign text of 50 campaigns was reviewed by both authors. After this review, the authors met and identified 6 campaign goals related to vaccines: 1) Helping with the costs of accessing vaccines (Access); 2) Creating facilities or spaces for people who do not wish to be vaccinated (Spaces); 3) Addressing loss of income or privileges due to being unvaccinated (Unvaccinated Individuals); 4) Seeking to provide the public information about vaccines (Advocacy); 5) Paying for legal actions or advocacy against vaccine mandates (Anti-Mandate); and 6) Responding to the consequences of a perceived vaccine injury or other adverse response to vaccination (Vaccine Injury). This review also identified two cross-cutting themes around how the need to fundraise is often justified: 1) Language invoking freedom, autonomy, consent, or human rights; and 2) Religious justifications. The first author then reviewed the campaign text for all campaigns and excluded any campaigns that did not seek funding related to vaccination for humans or where vaccination was not part of the aim of the campaign. Excerpts relating to the two themes were extracted from included campaigns. Each campaign was assigned one or more types and identified as relating or not to the COVID-19 pandemic. The second author then reviewed 10% of the campaigns to ensure agreement in how campaign types were assigned and themes interpreted. Any disagreements were resolved through discussion among the two authors.

## Findings

### General campaign characteristics

This process identified 765 crowdfunding campaigns hosted on the GiveSendGo crowdfunding platform that sought financial support related to vaccines or vaccination in humans. These campaigns raised $6,814,817 (median $78.80) out of a requested $838,578,249 (median $15,000) from 74,927 (median 1) donors. The campaigns were shared online 15,487 (median 0) times and received 49,670 (median 2) ‘prayers’–a signifier of support akin to a ‘like’ button on other crowdfunding platforms that also may include a message to the campaigner. 312 (40.8%) of the campaigns raised no money. 154 campaigns did not post a fundraising goal. Of the 611 campaigns that did have a fundraising goal, 22 (3.6%) met or exceeded it.

12 (1.6%) campaigns collected over $100,000 each, totalling $4,521,217 or 66.3% of all funding raised. These most successful campaigns included a legal fund to challenge COVID-19 vaccine mandates in Australia ($471,532), $471,926 for billboards in the US warning of the danger of vaccines for COVID-19, $474,783 to support a ‘whistleblower’ warning of the dangers of a specific COVID-19 vaccine, $781,446 for a protest against COVID-19 vaccine mandates in Canada, and $788,690 for a legal fund for US-based airline employees fighting a COVID-19 vaccine mandate.

615 (80.4%) of these campaigns were from the US, followed by 101 (13.2%) from Canada, 18 each (2.4%) from Australia and the European Union, 10 (1.3%) from the United Kingdom, and 1 each from Japan, New Zealand, and South Africa. 698 (91.2%) of the campaigns focused on vaccines for COVID-19 and 67 (8.8%) discussed other vaccine types. The most common category of crowdfunding campaign was Anti-Mandate (n = 346, 44.2%), followed by Unvaccinated Individuals (n = 226, 28.9%), Vaccine Injuries (n = 84, 10.7%), Advocacy (n = 76, 9.7%), Access (n = 34, 4.3%), and Spaces (n = 17, 2.2%). 18 campaigns were placed in two categories. Table 1 presents the number of campaigns, funding received, goal, donors, and shares for each campaign type. US campaigns had their highest overall proportion among Access campaigns and Canadian campaigns were proportionally greatest in Spaces campaigns.

**Table 1:** Campaign types.

	Number of Campaigns	Raised Total	Raised Median	Goal Total	Goal Median	Donors Total	Donors Median	Shares Total	Shares Median	No Donors (%)	Covid-related %
**Anti-Mandate**	346	$4,188,943	$225	$794,893,648	$23,214	28,151	3	6,831	1	33.5%	99.1%
**Unvaccinated Individual**	226	$546,689	$0	$11,182,114	$10,000	5349	0	1737	0	56.2%	99.6%
**Vaccine Injuries**	84	$232,544	$78.20	$3,362,733	$15,000	3122	2	958	1	36.9%	82.4%
**Advocacy**	76	$1,919,515	$170	$12,817,071	$15,000	38,970	2.5	6,138	0	36.8%	80.3
**Access**	34	$50,346	$270	$233,067	$2,900	322	3	68	0	32.4%	0%
**Spaces**	17	$14,693	$8.03	$17,056,060	$30,000	148	1	102	0	38.9%	100%

Table 2 presents themes by the campaigner’s location.

**Table 2:** Campaign types and location.

	US Total	US %	Canada Total	Canada %	Other Total	Other %
**All Types**	615	80.4%	101	13.2%	49	6.4%
**Anti-Mandate**	281	81.2%	45	13.0%	20	5.8%
**Unvaccinated Individual**	176	77.9%	37	16.4%	13	5.7%
**Vaccine Injuries**	64	76.2%	13	15.5%	7	8.3%
**Advocacy**	59	77.6%	10	13.2%	7	9.2%
**Access**	32	94.1%	0	0.0%	2	5.9%
**Spaces**	13	76.5%	4	23.5%	0	0.0%

**Fig 1:**
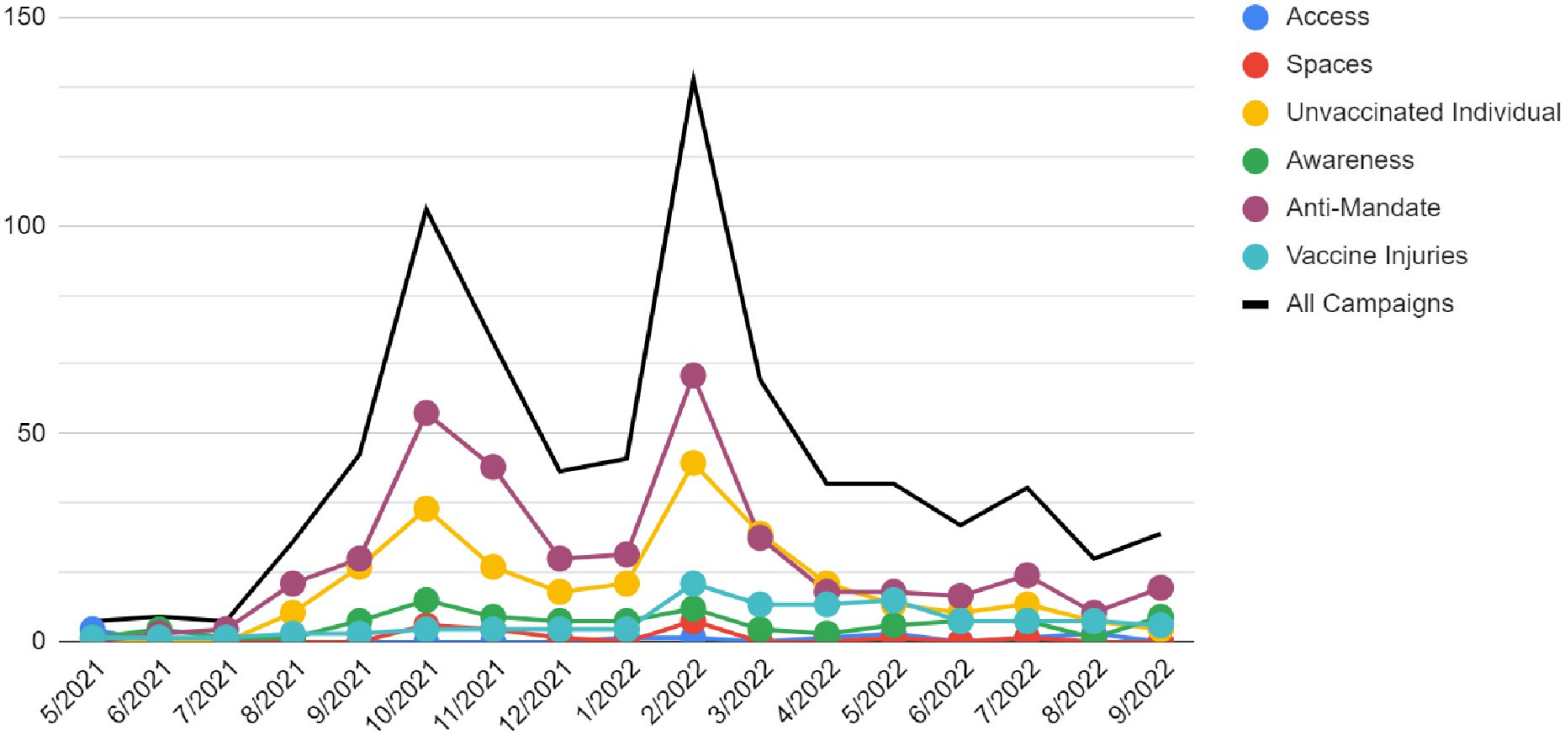
Vaccine crowdfunding campaigns over time.

These campaigns were initiated between March 24, 2016 and September 30, 2022. Of the 745 campaigns where initiation dates could be determined, 715 (96.0%) were initiated on or after May 1, 2021. These 30 earlier campaigns skewed toward the Access category (n = 23, 76.7%). Of the remaining campaigns, Access campaigns occurred at a low but consistent rate after May 1. The other campaigns saw spikes of activity around October 2021 and February 2022 with the exception of Vaccine Injury campaigns only spiking in October 2021. Fig 1 depicts crowdfunding campaign types over time and Table 3 shows campaign types by month.

**Table 3:** Campaign types by month.

	5/21	6/21	7/21	8/21	9/21	10/21	11/21	12/21	1/22	2/22	3/22	4/22	5/22	6/22	7/22	8/22	9/22
**Anti-mandate**	0	2	3	14	20	55	42	20	21	64	25	12	12	11	16	7	13
**Unvaccinated Individual**	0	0	0	7	18	32	18	12	14	43	26	14	9	7	9	5	3
**Vaccine Injuries**	1	1	1	2	2	3	3	3	3	14	9	9	10	5	5	5	4
**Awareness**	1	3	1	1	5	10	6	5	5	8	3	2	4	5	5	1	6
**Access**	3	0	0	0	0	0	0	0	1	1	0	1	2	0	1	2	0
**Spaces**	0	0	0	0	0	4	3	1	0	5	0	0	1	0	1	0	0
**Total**	5	6	5	24	45	104	72	41	44	135	63	38	38	28	37	20	26

### Campaign characteristics by goal types

Of Anti-Mandate campaigns, 343 (99.1%) were directed at COVID-19 vaccine mandates. These campaigns could target all vaccine mandates or a specific mandate affecting a target group. 130 (37.6%) of these campaigns targeted COVID-19 mandates generally. Campaigns targeting mandates generally were inspired by the anti-mandate Freedom Convoy protest in Canada and sought to support or emulate that protest. Other general Anti-Mandate campaigns included proposals to bring legal cases against all mandates in a specific political or geographic jurisdiction such as a state or country. All but one of the remaining campaigns (n = 216, 62.4%) targeted specific vaccine mandates. These targets were typically particular employers, including in the medical system (n = 46), other private employers (n = 31), government employers at the municipal, state/provincial, and federal levels (n = 22), employers of first responders such as police officers and firefighters (n = 18), the military (n = 18), and airlines (n = 8). Other campaigns focused on vaccine mandates in educational environments affecting primary, secondary, and post-secondary students, teachers, and support staff (n = 53), court-ordered mandates for children or incapacitated adults, typically as part of a child custody dispute (n = 13), and children generally (n = 7). Campaigns with specific targets were considerably more effective (median $550 from median 5.5 donors) than campaigns with general aims (median $8.92 from median 1 donor).

Unvaccinated Individual campaigns were motivated by the loss of income (199, 88.1%) and access to services (n = 27, 11.9%). In the former group, campaigners typically sought to aid individuals or groups who were struggling to pay for basic necessities due to loss of income. The most common employment categories in this group were medical personnel (n = 46), educators (n = 18), first responders (n = 15), military personnel (n = 15), and government employees (n = 11). Campaign recipients experiencing a loss of income ranged from individuals seeking to survive month to month and “prevent hunger and homelessness while figuring out how to manage without income” to recipients who were seeking help with a change of career after leaving a job with a vaccine mandate. Loss of services included loss of access to education, the inability to travel domestically or abroad, and loss of access to medical treatment. In some cases, loss of access to medical treatment could include potentially life-threatening consequences as with campaigns for individuals who were refused organ transplantation due to being unvaccinated.

Vaccine Injury campaigns generally sought help paying for medical bills and loss of income resulting from perceived vaccine injuries and for advocacy for people harmed by vaccines. In campaigns not related to COVID-19 vaccines, two thirds of all recipients (n = 10) were minors. These campaigners typically sought help with treatments for autism spectrum disorder, which campaigners stated was a result of childhood vaccines. Adults describing injuries from non-COVID-19 vaccines were more varied and included skin damage, chronic fatigue syndrome, and paralysis. Among campaigners seeking help for injuries perceived to have resulted from vaccines against COVID-19, recipients were typically adults. Specific adverse reactions and injuries said to stem from vaccines for COVID-19 included cardiovascular disease, autoimmune disease, kidney damage, neurological disease, generalized fatigue, cancer, and death. Causality between COVID-19 vaccines and injury was usually established by chronological proximity between vaccination and the injury, where the campaigner noted good health prior to being vaccinated or new symptoms post-vaccination. A typical story would describe how a “perfectly healthy patient just got vaccinated and suddenly couldn’t function”. Certainty of this causation ranged from the campaigner stating they were told a vaccine was definitely the cause, to having “every reason to believe that it was from the vaccine”, to the proximity of harm being “a bit suspicious”. These campaigns frequently included specific descriptions of COVID-19 vaccines as dangerous, including calling them “leaky”, stressing the “experimental” nature of MRNA vaccines, and disputing whether they were in fact vaccines at all by using quotation marks around “vaccine”. In some cases, campaigners delved into conspiracy theories about the ingredients and outcomes of these vaccines including claims that they contain “graphene oxide, parasites, nano-bots” and had killed “between 20,000 to 200,000” in the UK. COVID-related campaigns were more successful than non-COVID campaigns with 66.2% of the former receiving at least 1 donation (median $100 from median 2 donors) as compared to half of non-COVID campaigns receiving support (median $0 from median 0 donors).

Advocacy campaigns generally sought to inform the public about what the campaigner perceived as the dangers of vaccinations. These projects included a weekly print newspaper seeking to publish stories about vaccine adverse events among African Americans, video equipment for producing videos about “all positives and negatives about each vaccine”, billboards warning about vaccine side effects, interviews with people experiencing vaccine injuries, and online communities for the discussion of the negative aspects of vaccines. These campaigns frequently expressed frustration with the “main stream media” which was failing to report on stories about vaccine injuries because they “push propaganda for the government and the agenda of the elitist”. Advocacy campaigns were often tied to related goals to highlight what were perceived as “censored” stories concerning election fraud and the dangers of racial justice movements such as Black Lives Matter. In doing so, they regularly referenced the QAnon conspiracy movement, “World Economic Forums globalist agenda”, and “New World Order and the Satanists in power in DC and abroad”, dismissed the COVID-19 pandemic as a “Plandemic or Scamdemic”, and appealed to the support of “patriots” who were able to see through these forces. Through pursuing this web of interconnected stories, some campaigners noted having been banned or restricted on mainstream outlets such as YouTube, Facebook, and GoFundMe. Advocacy campaigns related to COVID-19 vaccines were more successful (median $202.50 from median 3 donors, 32.8% raising no money) than other vaccine advocacy campaigns (median $0 from median 0 donors, 53.3% raising no money).

Access campaigns took a very different view of the value of these interventions than other campaign types. None of these campaigns sought access to vaccines for COVID-19. In most cases (n = 25), they were part of a larger campaign to pay for travel expenses, usually connected with a religious mission or educational opportunity abroad. Destinations were most commonly in Africa (n = 14), followed by the Americas (n = 8), Australia (n = 2) and Asia (n = 1). These campaigns were typically matter of fact about the value of vaccines, listing them as “required vaccines” that were necessary for the trip along with funding for airfare, visas, and accommodations. Others (n = 8) sought to provide vaccines to groups with limited access to health services. The most common target communities for these campaigns were in Africa (n = 4), the Americas (n = 2), Asia (n = 1), and Europe (n = 1). These campaigns motivated giving based on the benefits of vaccines for protecting the health of individuals as with a campaign for people in the Niger Delta region where “many vulnerable young people are unable to access vaccinations, operations and health care support that they need to survive”. One other campaign sought to promote vaccine research, specifically for a “Personalized Vaccine Trial” for an American child with brain cancer.

Finally, Spaces campaigns were all focused on providing facilities for people who chose not to be vaccinated against COVID-19. These campaigns sought to fund a school “for children to learn in an environment where they could get an education without wearing a mask and worrying about getting a vaccination that could harm them”; an ice-skating rink for unvaccinated people; a “Natural Law Trust” hospital that would “not have to comply with the CDC and WHO guidelines”; an airline for the unvaccinated; and a “Conservative Christian community”, among others. Motivations for creating these spaces included protecting users from feeling pressured to receive a COVID-19 vaccine or providing alternatives for people who could not participate in certain activities due to being unvaccinated. More modest and purpose-suited projects tended to center on reactions to vaccine mandates and other pandemic restrictions. More expansive projects for alternative communities were often meant to suit a larger set of religious and cultural goals than solely being unvaccinated, including teaching children “inconsistencies in the theory of evolution” and providing protection from “BLM and riots or perseuction [sic] of trump voters”.

### Cross-cutting themes

Two themes cut across these campaign categories. First, many campaigners saw their crowdfunding goals as furthering freedom in some form. Typically, these campaigners felt they had faced or were facing coercion to become vaccinated, often from a “tyrannical” government or employer who engaged in “Medical Fascism” or “Marxist ideologies and indoctrination”. These fundraisers could help respond to this coercion through advocacy and legal means or be used to address the harms of being coerced to become vaccinated. A variety of labels were used to describe the values associated with personal choice to become vaccinated, including “freewill”, “individual choice”, “personal choice”, “liberty”, “personal autonomy”, and “freedom”. Others referred to legal and natural rights that granted individual freedoms using terms including “civil liberties”, “human rights”, and “Natural Law” or referred to specific legal frameworks including “international treaties”, the “Nuremberg codes”, and the US constitution. Some of this language focused on the medical aspect of vaccination and borrowed language from bioethics (“bodily autonomy”, “right to refuse medical treatments”, “informed consent”, “bodily integrity”) and reproductive freedom movements (“Pro-Choice”, “My Body, My Choice”). Campaigns seeking access to vaccines did not highlight the coercive element of vaccine promotion campaigns; rather, they described vaccines as enabling better health or allowing campaigners to spread religious messages such as that “freedom is obtainable through Christ”.

A second theme found across these categories was religious (usually Christian) faith. References to religion were frequently tied to the religious identity of the campaigner (“Christian mother”; “a follower of Jesus”) or used to motivate giving (“We are appealing for all whose hearts the Lord will touch to contribute”) and thank donors and other supporters (“God bless you all”). In other cases, the campaigners’ religious beliefs were the basis on which they sought to avoid vaccination. Religious objections to COVID-19 vaccines in particular were often left unclear, with the campaigner instead focusing on the difficulty of proving a religious objection (“[employer] sent me 9 additional questions that were extremely invasive”), outrage at having been denied an exemption (“Many of these exemptions were ILLEGALLY Denied”), or sense of being discriminated against on the basis of religion (“religious discrimination by our employers by not honoring the religious exemptions”). When specifics about the religious objection to COVID-19 vaccines were given, they were typically tied to claims that the vaccines relied on the tissues of aborted fetuses (“Fetal cells were used in the production”; “desecration of the baby’s remains”) or religious prohibitions against self-harm where the vaccine was seen as dangerous. For campaigners objecting to being vaccinated on religious grounds, the two themes often overlapped as they saw coercive pressure to be a violation of “Our rights, religious freedoms and bodily autonomy”.

## Discussion

### Relatively successful crowdfunding on GiveSendGo

This study identified campaigns related to vaccines and vaccination on the GiveSendGo crowdfunding platform. The large majority of these campaigns were initiated during the COVID-19 pandemic and related to COVID-19 (91.2%). COVID-19-related campaigns were more successful in attracting donations than non-COVID-19 campaigns among Unvaccinated Individual and Advocacy campaigns. Anti-Mandate campaigns raised the highest median amount ($225) whereas Unvaccinated Individual campaigns fared the worst (median $0) even though Unvaccinated Individual campaign recipients often faced a job loss or loss of services due to vaccine mandates. This suggests that campaigns pursuing political and policy ends were more appealing to donors than campaigns for the individuals impacted by these policies. Anti-Mandate campaigns may also have benefitted from including children as beneficiaries as crowdfunding campaigns for children often outperform those for adults [17].

The campaigns in this study had larger median goals ($15,000) than COVID-19-related campaigns on GoFundMe analyzed by Igra et al. [4] and Saleh et al. [1] (both $5,000) and Covid-19 vaccine-related campaigns on GoFundMe [18] (median $10,000). The campaigns in this study were more successful in terms of median funding raised ($78.80 vs. $65) and percentage receiving donations (59.2% vs. 56.8%) than those in Igra et al’s [4] analysis of COVID-19-related campaigns on GoFundMe. However, Saleh et al. [1] found much higher support in early COVID-19-related campaign on the GoFundMe platform with these campaigns raising median $930. The campaigns in this study raised less money than in Snyder et al’s [18] analysis of COVID-19 vaccine-related campaigns on GoFundMe (median $921) where 52.1% of campaigns received support.

### Attitudes toward vaccines

Access campaigns were an outlier among the campaign types in this study in that they saw vaccines as necessary or desirable. They also raised the highest median amount ($270) though they were often part of a larger campaign to support religious proselytizing, religious education, or medical needs. In these campaigns, seeking access to vaccines seemingly did not negatively impact fundraising and campaigners did not feel a need to excuse fundraising for vaccine access. This could be because of an othering dimension within these campaigns: the recipients were typically either residents of lower income settings or residents of high-income settings seeking to travel to a low-income community with higher exposure to tropical diseases. Moreover, none of these campaigns were for vaccines related to COVID-19 and they often predated the pandemic, thus escaping the politicization of vaccines associated with the pandemic.

This study demonstrates that crowdfunding campaigns can provide meaningful information about how the public responds to public health messaging and interventions, how misinformation spreads online, and how political movements and news media influence public perceptions about public health measures. Among campaigns related to COVID-19 vaccines on the GiveSendGo platform, the language used to appeal to potential donors was often heavily politicized and included misinformation about vaccine safety and conspiracy theories about the motivations behind public health actions. This narrative was frequently presented in terms of oppression and victimization of the campaign recipient where national and international political and economic forces worked to coerce individuals into being vaccinated against their wishes and, often, religious beliefs. Signalling through text, images, and videos is effective in communicating to potential donors that their values align with those of the campaigner [19]. These narratives about COVID-19 vaccines can be contrasted with vaccine-related campaigns on the GoFundMe platform [18]. These latter campaigns ranged from valuing vaccines as bringing an end to the pandemic to seeing them as having less clearly positive but not harmful outcomes.

### The impact of gatekeeping

The rate of campaign creation in this study surged in October 2021 and February 2022. While the causes of these surges are not clear from the data collected, they may be connected to events that drew attention to the GiveSendGo crowdfunding platform rather than changes in COVID-19 infection rates or vaccine mandates and other public health actions. In October 2021, GoFundMe reiterated its policy against campaigns that promote vaccine misinformation [7] and received press coverage for removing a fundraiser for an anti-vaccine mandate lawsuit that had raised $180,000 (and then moved to GiveSendGo) [20, 21]. In February 2022, the massively successful anti-mandate Freedom Convoy fundraiser gained traction on GoFundMe, was banned on that platform, and moved to GiveSendGo, attracting massive press attention along the way [22, 23].

These surges in campaign activity demonstrate the larger effects of gatekeeping activities by crowdfunding platforms. Restrictions on misinformation on other social media platforms such as Twitter, Facebook, and Instagram have seen some success as these platforms rely on large numbers of active users to be successful and advertisers for revenue. These network effects make it more difficult for alternative platforms with more permissive moderation standards to succeed [24]. However, GiveSendGo has demonstrated that crowdfunding campaigns that have been restricted on mainstream platforms can find success elsewhere—in fact, these restrictions seem to bolster the number of campaigns and donors on the GiveSendGo platform. These restrictions draw attention and sufficient donors to alternative platforms for crowdfunding campaigns to be relatively successful in the terms that count in crowdfunding—financial support. Moreover, this activity generates benefits to the crowdfunding platform itself as they typically rely on contributions or ‘tips’ from donors rather than money from advertisers that may be driven away by lax moderation [25]. GiveSendGo appears to be well aware of this phenomenon as they have issued press releases [26, 27] and appeared on traditional media outlets following restrictions to campaigns on the GoFundMe platform [5].

### Limitations

This study faced several limitations. The search terms used (“vaccine” and “vaccination”) will not have identified some campaigns relevant to the study, including those using informal terms for vaccination (“vax”, “jab”) and those making clear reference to vaccination without using that or similar words (“the mandates”). Some relevant campaigns that included “vaccine” or “vaccination” in their campaign text were removed from GiveSendGo prior to data collection. This included, for example, the Freedom Convoy campaign that raised nearly $10 million in February, 2022. Older campaigns are more likely to have been removed than newer campaigns and levels of donor support may also influence the decision to remove a campaign. As a result, this study presents an incomplete set of vaccine-related crowdfunding activity on the GiveSendGo website and understates the number of campaigns and the funding raised by them.

## Conclusions

This study examined crowdfunding activity linked to vaccines on the GiveSendGo crowdfunding platform, including a surge of crowdfunding activity from mid-2021 to late 2022. Most of these campaigns reflect political narratives about the dangers of vaccines and public health responses to the COVID-19 pandemic. These narratives give reason for concern about public attitudes about vaccines going forward as they both spread these viewpoints online and directly fund anti-vaccine messages and initiatives. These campaigns also demonstrate the challenges of content moderation and banning campaigns containing vaccine misinformation—moderation by one platform can be used as a business opportunity by another. Specifically, during the COVID-19 pandemic, vaccine-related campaigns banned on one platform were seemingly able to find success, by the standards of donation-based crowdfunding, elsewhere.

The findings in this study may apply to crowdfunding for other causes that have been removed from mainstream platforms including campaigns for people accused of violence, campaigns encouraging hate and discrimination, and other campaigns spreading misinformation. Insofar as these campaigns were successful due to rather than despite their politically charged messages about vaccines, other charged or ‘loud’ crowdfunding campaigns may experience considerable success [28]. There is emerging evidence that less restrictive crowdfunding platforms like GiveSendGo are a significant source of financial support for political extremists like the Proud Boys, white supremacist groups, insurrections against democratically elected governments, anti-LGBTQ+ organizations, and political conspiracy movements like QAnon [29]. Study of crowdfunding campaigns related to these other movements would help to determine which of these causes are most successful and whether restrictions on mainstream crowdfunding platforms drive use of less restrictive platforms as in the case of vaccine-related campaigning. If gatekeeping by mainstream crowdfunding platforms is not effective in reducing the numbers and success of campaigns financing political extremism, hate, and misinformation, then these findings would support more direct actions by regulators. Thus, continued study of the full range of crowdfunding platforms is warranted.
